# Aerobic physical exercise improves quality of life in temporal lobe epilepsy

**DOI:** 10.1055/s-0045-1804919

**Published:** 2025-03-19

**Authors:** Shai Oisiovici Laks, Nathalia Volpato, Nikolas Coelho, Mateus Henrique Nogueira, Pamela Regina Henning, Aline de Fatima Dias, William Souza Martins Ferreira, Clarissa Lin Yasuda, Luciana Ramalho Pimentel-Silva, Fernando Cendes

**Affiliations:** 1 Universidade Estadual de Campinas, Faculdade de Ciências Médicas, Campinas SP, Brazil.

**Keywords:** Epilepsy, Temporal Lobe, Quality of Life, Exercise, Seizure

## Abstract

**Background** A prior study showed that people with temporal lobe epilepsy (TLE) with an active lifestyle present a better quality of life (QoL), but the influence of aerobic physical exercise (APE) in the QoL of these patients is still unclear. As pharmacoresistance is commonly associated with TLE, the possibility of seizures during the activities might prevent patients from benefiting from APE.

**Objective** To analyze the influence of APE in the QoL of TLE patients and also focus on the seizure worry subitem of the QoL in epilepsy 31 (QoLIE-31) questionnaire.

**Methods** We analyzed data from 25 individuals with TLE who participated in a 6-month APE program under the supervision of a board-certified physical trainer. TLE patients were divided into training (TLE-training) and control (TLE-control) groups. The TLE-training group underwent an APE program of 6 months, while the control group was oriented to keep their routine. We assessed all patients with the QoLIE-31 at baseline and after 6 months. We used a mixed-between-subjects ANOVA to assess the APE intervention compared with TLE-control pre- and postintervention on the QoL.

**Results** We found a significant interaction between intervention groups and APE-time ( *p* = 0.0005), showing that only patients in the TLE-training group presented higher QoL after the intervention ( *p* = 0.001). We found no significant differences between groups for seizure worry ( *p* = 0.50).

**Conclusion** There was improvement in QoL due to APE, with no increase in seizure worry, which might be a feature of concern for both patients and healthcare practitioners. Further studies should focus on long-term interventions to evaluate the impact of APE in QoL.

## INTRODUCTION

Epilepsy is a chronic neurological disorder that affects approximately 1% of the world's population. 1 The clinical diagnosis of epilepsy defined by the International League against Epilepsy (ILAE) is based on the occurrence of any of the following: at least two unprovoked epileptic seizures in an interval of more than 24 hours; one unprovoked (or reflex) seizure and a probability of further seizures similar to the general recurrence risk (at least 60%) after two unprovoked seizures, occurring over the next 10 years; identification of an epileptic syndrome. 2 4 The seizures are characterized by episodes of hyperactivity of the neuronal circuits of the brain, generating excessive or synchronous abnormal electrical discharges in specific neuronal groups. 2

Epilepsy may be associated with several neurological disorders, such as cognitive decline, and psychiatric disorders, including anxiety and depression, which go far beyond the consequences of seizures and impact quality of life (QoL), constituting specific epileptic syndromes. 3 4 Such comorbidities result in a reduction of the QoL of patients with epilepsy in addition to the stigmatization and lack of understanding about the disease, which often prevents the adequate treatment of this population. The severity of this situation is represented by the fact that 75% of people with active epilepsy do not receive adequate treatment, a number that is mostly concentrated in countries with medium and low per capita income. 5

Epilepsy can be classified as focal (affecting a part of the brain), generalized (involving large areas of both cerebral hemispheres), combined (patients who present both types of seizures), or unknown. 3 Temporal lobe epilepsy (TLE) stands out as the most frequent focal epilepsy in adults, which is often pharmacoresistant. 6 In mesial temporal lobe epilepsy, the most common subtype, seizures begin in the mesial structures, mainly in the hippocampus (with or without the presence of hippocampal sclerosis). 7 Seizures in TLE may also originate in the anterior or lateral temporal cortex; 6 as this disease is known to affect the neural network, with structural 8 and functional alterations that go beyond the temporal lobe. 9

Aerobic exercise performed with ideal regularity, intensity and frequency promotes specific processes of morphological, biochemical and physiological adaptation that lead to the improvement of functional capacity, which is widely measured by peak oxygen consumption (VO2) and health in general. 10 11 The benefits of physical exercise, specifically aerobic physical exercise (APE), on systemic health and QoL in general are already well-known and documented in the literature. 10 However, the application of APE to neurological diseases and neurobiological adaptations is still poorly understood.

In this context, the effects of APE on TLE and other epilepsies, in general, are not well known. Studies in this area have been performed in recent years and have shown significant results in people with epilepsy after intervention with an exercise program. Among the benefits, the following stand out: improved physical capacity, general health and psychological state. 12 13 A reduction in the frequency of seizures has also been observed in some specific cases and animal models. 14 15 In addition, people with TLE who remain physically active have higher QoL scores. It is also essential to include a cardiorespiratory assessment in the methodological aspects to assess the levels of physical activity performed by people with epilepsy since the indirect assessment, through scales such as the International Physical Activity Questionnaire – IPAQ, 16 may present inconsistencies. 11

Despite the possible benefits, people with epilepsy generally have reduced rates of APE, which has been associated with several studies because of fear of seizures during activity (which could lead to injuries), side effects of antiseizure medications, and psychosocial problems, such as difficulties in mobility and low motivation. 17 The fear of having seizures, a matter of concern for both patients and healthcare practitioners, can be analyzed by the QoL in Epilepsy 31 (QoLIE-31) inventory, a validated questionnaire with a seizure worry evaluation subitem. It is still not known whether the performance of APE can act as a worsening factor for seizures since most studies on this matter are case reports requiring a better investigation. 18 19

In a previous non-interventional study that included 38 patients with TLE and 20 healthy controls, we found that physically active patients had a better QoL than those who were inactive. Furthermore, the controls had better physical capacity than patients with TLE by cardiopulmonary effort test. 11 In the present study, we investigate if applying an APE program on TLE patients can improve their QoL without increasing their seizure worry and whether it can be considered a possible non-pharmacological intervention in this matter.

## METHODS

This study is a controlled clinical trial approved by the Ethics and Research Committee of the Universidade Estadual de Campinas (UNICAMP), with the protocol number CAAE 39547214.0.0000.5404. The objective was to assess the influence of APE on the QoL of TLE patients using the QoLIE-31, focusing on the seizure worry subitem.

We recruited 25 individuals with TLE at UNICAMP's Teaching Hospital. All patients were diagnosed based on comprehensive clinical evaluation, including a detailed history, general and neurological exams, serial electroencephalograms (EEGs), and magnetic resonance imaging (MRI). During the recruitment, we excluded subjects that presented any of the following factors:

- Previous neurological surgery;
- Delayed neuropsychomotor development diagnosed by neuropsychological evaluation;
- Presence of other possibly epileptogenic lesions (such as calcifications, extratemporal focal cortical dysplasia, and tumors);
- Ongoing infectious or inflammatory disease resolved in less than 1 month of data collection; and
- Inability to provide free and informed consent.

Patients were divided into the TLE-training and -control groups. The TLE-training group went through a 6-month APE program, while the control was oriented to keep their routine. The program consisted of walking exercises at an individualized aerobic intensity, with two fixed weekly sessions, of a maximum of 60 minutes, for 16 weeks.

Both groups were assessed at baseline and after 6 months by QoLIE-31, which is a reputed instrument to evaluate the QoL in subjects with epilepsy. The questionnaire ranges from a 0 to 100 score, in which a higher mark represents better perceived overall wellness, social functioning, and cognitive performance. It includes seven main topics that encompass a general notion of the experience of life dealing with epilepsy: seizure worry, overall QoL, emotional well-being, energy/fatigue, cognitive, medication effects, and social function. 20 We analyzed the general score of the test and each main topic individually.

As for the effectiveness of the APE program, we assessed patients at the same time margin as the QoL, using a maximal effort cardiopulmonary exam on a treadmill. We monitored patients with an electrocardiogram and an Oxycon Pro gas analyzer (Erich Jaeger GmbH, Hoechberg, Germany), which evaluated oxygen consumption and CO2 production. Aerobic power was expressed as the VO _2Lmax_ , considering the average values obtained during the last 30 seconds of the cardiorespiratory evaluation. 11 21 Finally, we reviewed medical charts to obtain the seizure frequency up to 1 year prior and after the intervention period for both groups.

Categorical data were analyzed using the χ ^2^ test or Fisher's exact test. We evaluated differences in age using the *t* -test. The statistical analysis consisted of a mixed-between-within subjects' analysis of variance (ANOVA) to investigate the impact of the APE intervention compared with TLE-control pre- and postintervention on the QoL in general and in each specific parameter of the QoLIE-31. We tested aerobic physical capacity using an analysis of covariance (ANCOVA), including pre-intervention VO _2Lmax_ level as a covariate (to minimize possible initial variations between groups). Because the normality and the homogeneity of variance for the ANOVA were not assumed (given that seizure frequency most likely follows a Poisson distribution, i.e., count data) neither for the data nor for the residuals, we applied a separate Friedman ANOVA for nonparametric paired data for each group to test whether there were changes into the frequency seizure before or after the APE program. We presented data as mean ± standard deviation (SD) or frequency and percentage [n (%)]. We set *p* < 0.05 as statistically significant. All statistical analyses were performed using the Statistical Package Social Sciences (SPSS, IBM Corp., Armonk, NY, USA), version 29.

## RESULTS

Both TLE-control and training groups were matched for baseline clinical and demographics data, except for baseline seizure frequency ( Table 1 ).

**Table 1:** Descriptive statistics on the TLE-training and control groups

		Control ( *n* = 14)	Training ( *n* = 11)	Test statistics	*p-* value
Age (years)		49.1 ± 5.7	50.3 ± 5.7	t (23) = −0.52	0.6
Sex	Female	7(50%)	8 (72.7%)	χ ^2^ (1) = 1.33	0.4
	Male	7 (50%)	3 (27.3%)		
Epilepsy onset (years)		12.1 ± 11.9	13.4 ± 6.1	t (23) = −0.31	0.76
Epilepsy duration (years)		36.9 ± 13.0	36.9 ± 8.1	t (23) = −0.004	0.9
Lateralization	Right	6 (42.9%)	4 (36.4%)	χ ^2^ (2) = 0.38	0.83
	Left	6 (42.9%)	6 (54.5%)		
	Bilateral	2 (14.3%)	1 (9.1%)		
Seizure frequency (monthly, at baseline)		1 (0–4)	2 (0–9)	U (24) = 105.5	0.047

Abbreviation: TLE, temporal lobe epilepsy.

The APE resulted in an increase of VO _2Lmax_ and VO _2L/Kg_ ( *p* = 0.008 for both variables) and a decrease in heart rate (HR _max_ , *p* = 0.009) postintervention in the TLE-training only, demonstrating that the training was effective, while these parameters did not change between baseline and after 6 months in the control group.

**Figure 1:**
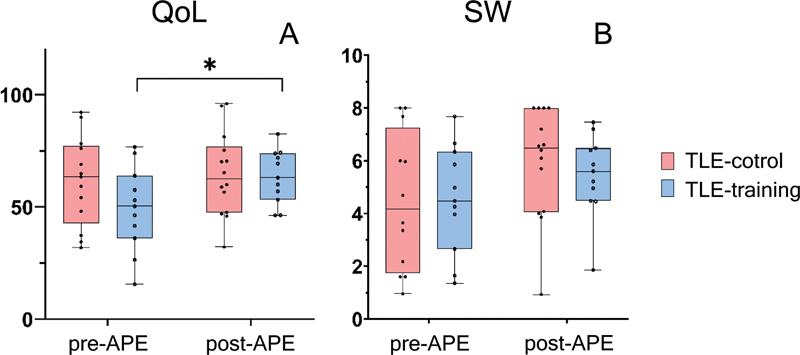
Abbreviations: APE, aerobic physical exercise; QoL, quality of life; SW, seizure worry. Note: * *p* = 0.01. Box plot showing the interactions and main effects of APE groups on the ( **A** ) QoL and ( **B** ) SW scores pre- and post-APE. Error bars represent the 95% confidence interval.

Regarding QoL, we found no significant difference between groups ( *p* = 0.55), and a significant effect of APE ( *p* = 0.001). However, there was also a significant interaction between intervention-groups and APE-time ( *p* < 0.0005), showing that only patients in the TLE-training group presented with higher QoL after the intervention ( *p* = 0.01), as shown in Figure 1A , while TLE-control did not show any significant differences pre- and post-APE ( *p* = 0.6).

As for seizure worry, we found no significant differences between groups ( *p* = 0.5), as well as no significant differences in APE-time effects ( *p* = 0.95) or interaction ( *p* = 0.11, Figure 1B , Table 2 ). Finally, we found that the seizure frequency did not change significantly after the APE intervention, neither for the control-TLE group (Χ ^2^ [1] = 0.2, *p* = 0.65) nor for the training-TLE (Χ ^2^ [1] =0.67, *p* = 0.41). The control-TLE presented with a 1-year postintervention seizure frequency (median [range]) of 1 (0–3) seizures while the training-TLE presented with 2 (0–30) seizures.

**Table 2:** Test statistics and effect sizes for group effects, APE effects, and interactions for key variables

	Group effect		APE effect		Interaction
	Test statistic	Effect size	Test statistic	Effect size	
VO _2Lmax_	F _1, 20_ = 1.08	0.051	F _1, 20_ = 4.48	**0.183**	F _1, 20_ = 5.81
VO _2L/Kg_	F _1, 20_ = 1.67	0.077	F _1, 20_ = 1.85	**0.085**	F _1, 20_ = 5.36
QOL	F _1, 49_ = 0.36	0.007	F _1, 49_ = 11.83	**0.195**	F _1, 49_ = 15.97
SW	F _1, 49_ = 0.47	**0.010**	F _1, 49_ = 0.004	0.0001	F _1, 49_ = 2.57

Abbreviations:  , partial eta squared; APE, aerobic progressive exercise; QOL, quality of life; SW, seizure worry; VO _2/Kg,_ relative aerobic capacity/power;VO _2Lmax,_ aerobic capacity/power.

Note: The largest effect size within each univariate model is indicated in boldface.

All the test statistics for the group and APE effects comparisons are presented in Table 2 .

## DISCUSSION

In this study, we successfully demonstrated that the aerobic physical exercise was capable of inducing a significant improvement in maximal aerobic capacity in subjects with epilepsy, thus indicating the APE program was effective. We also noted improved overall QoL only in the training group after 6 months, and no change in seizure concern.

In a previous study, we demonstrated that patients with TLE who were physically active had a better QoL than those who were sedentary using the WHOQoL-BREF questionnaire. 11 In the present study, we used the QoLIE-31 questionnaire in a controlled trial comparing a group before and after a 6-month APE and a control group without APE. Our results demonstrated that the effective APE improved QoL after the 6-month intervention in those patients undergoing aerobic training.

As for the seizure worry subitem of the QoLIE-31, we found no significant difference between pre- and postintervention, showing that the APE program did not increase seizure worry. This subitem might be an important feature of concern for both patients and healthcare practitioners, preventing people with epilepsy from engaging in a regular APE routine. Accordingly, despite the baseline difference, the seizure frequency did not change after the APE intervention period in either group. One patient in the training-TLE group had 9 seizures in the year prior to the intervention, increasing to 30 episodes 1 year postintervention. However, review of the medical charts indicated that this patient had a varying pattern with 30 seizures in the 2 years prior the intervention. This finding suggests that assisted APE did not exacerbate seizure frequency and expands previous findings 22 by evaluating longer follow-ups (up to 1 year pre- and postintervention). Although longer periods of aerobic exercise might be required to observe stronger or long-term effects on seizure frequency, a study has shown that even physical effort to exhaustion did not induce seizures in 17 individuals with TLE. 23

Our findings give support for longer interventions to evaluate the long-term impact of APE in the QoL of people with epilepsy. Additionally, it would be important to investigate whether more prolonged periods of APE can also help reduce seizure frequency. Along this line, experimental animal models of epilepsy have shown that physical exercise training programs can reduce the frequency and intensity of seizures. 14 21

In conclusion, our findings support the idea that individuals with epilepsy should be motivated and guided to engage in regular physical exercise at an appropriate intensity level to enhance their physical and emotional well-being.
